# A cost‐effectiveness threshold based on the marginal returns of cardiovascular hospital spending

**DOI:** 10.1002/hec.3831

**Published:** 2018-10-01

**Authors:** Pieter van Baal, Meg Perry‐Duxbury, Pieter Bakx, Matthijs Versteegh, Eddy van Doorslaer, Werner Brouwer

**Affiliations:** ^1^ Erasmus School of Health Policy and Management Erasmus University Rotterdam Rotterdam The Netherlands; ^2^ Institute for Medical Technology Assessment Erasmus University Rotterdam Rotterdam The Netherlands; ^3^ Department of Applied Economics Erasmus School of Economics Rotterdam The Netherlands

**Keywords:** Bayesian statistics, cost‐effectiveness analysis, opportunity costs, threshold

## Abstract

Traditionally, threshold levels of cost‐effectiveness have been derived from willingness‐to‐pay studies, indicating the consumption value of health (*v‐*thresholds). However, it has been argued that *v‐*thresholds need to be supplemented by so‐called *k‐*thresholds, which are based on the marginal returns to health care. The objective of this research is to estimate a *k‐*threshold based on the marginal returns to cardiovascular disease (CVD) hospital care in the Netherlands. To estimate a *k*‐threshold for hospital care on CVD, we proceed in two steps: First, we estimate the impact of hospital spending on mortality using a Bayesian regression modelling framework, using data on CVD mortality and CVD hospital spending by age and gender for the period 1994–2010. Second, we use life tables in combination with quality of life data to convert these estimates into a *k*‐threshold expressed in euros per quality‐adjusted life year gained. Our base case estimate resulted in an estimate of 41,000 per quality‐adjusted life year gained. In our sensitivity analyses, we illustrated how the incorporation of prior evidence into the estimation pushes estimates downwards. We conclude that our base case estimate of the *k*‐threshold may serve as a benchmark value for decision making in the Netherlands as well as for future research regarding *k‐*thresholds.

## 1 BACKGROUND

Over the years, cost‐effectiveness has gained a more prominent role in the reimbursement decision process for new technologies (Garber & Sculpher, 2011). Scientific output related to cost‐effectiveness research has also increased, with PubMed indexing 1,540 studies with “cost‐effectiveness” in the title in 2015 versus 582 studies in 2005. However, although considerable effort has been put into performing these studies and improving the analytical methods, some important questions have been less than sufficiently researched (Meltzer & Smith, 2011). One of these questions asks how we choose appropriate threshold levels for cost‐effectiveness ratios of new technologies. Consequently, we may know the ratio of costs to effects of an intervention, including advanced uncertainty analysis, but are left with very limited grounds to come to a societal decision on whether the ratio is acceptable or “too high.”

Traditionally, threshold levels of cost‐effectiveness have been derived from willingness‐to‐pay (WTP) studies (employing questionnaires), which have their roots in welfare economics (Ryen & Svensson, 2015). WTP‐based thresholds indicate the consumption value of health, can be interpreted as a demand‐side threshold, and are sometimes referred to as *v*‐thresholds (Claxton, Paulden, Gravelle, Brouwer, & Culyer, 2011; Claxton, Walker, Palmer, & Sculpher, 2010). More recently, it has been argued that threshold levels of cost‐effectiveness may also be obtained by estimating the marginal returns to health care using health spending data linked to health outcomes (Claxton et al., 2015; Claxton, Sculpher, Palmer, & Culyer, 2015; Vallejo‐Torres et al., 2016; Vallejo‐Torres, García‐Lorenzo, & Serrano‐Aguilar, 2018). The theoretical underpinning of this approach assumes that health care budgets are set exogenously and that implementing new health care technologies comes at a price of displacing other health care technologies. In other words, the cost of implementing new health care technologies equals the health foregone through the displacement of these other technologies—the health opportunity costs. These types of thresholds indicate the additional costs that would displace one quality‐adjusted life year (QALY) elsewhere in the health care system and are referred to as *k‐*thresholds or supply‐side thresholds (Claxton et al., 2011, 2010). Although in theory it would be most efficient to displace the least cost‐effective interventions and take the cost‐effectiveness of these interventions as a proxy for the *k‐*threshold, there are several problems with this approach. First, it is difficult to identify specific technologies that are displaced in practice. Second, for many currently covered interventions, the cost‐effectiveness is unknown. Third, it is likely that older technologies that are displaced by the new will not completely disappear; rather, the target groups for less specific technologies will be constrained or health care inputs such as labour will decrease as a consequence of implementing new technologies. Therefore, an estimate of the marginal returns to health care spending is the best available proxy for the threshold at which more health is displaced than gained. Such an estimate is an average of the cost‐effectiveness of all interventions that are displaced or expanded at the margin.

Under certain conditions and assumptions (i.e., in what economists refer to as the first‐best world), economic theory predicts that the *k*‐threshold equals the *v*‐threshold, as the marginal utility of consuming an additional euro in health care equals the marginal utility of consuming that euro outside health care (Meltzer & Smith, 2011). However, looking at the empirical literature, there is no reason to assume that in reality this is indeed the case. In their literature review of 24 WTP studies, Ryen and Svensson (2015) find that mean and median estimates for the WTP/*v*‐threshold of a QALY are €74,159 and €24,226, respectively, whereas the estimate of a *k*‐threshold based on marginal returns to health care, by Claxton, Martin, et al. (2015) found a value for England of £12,936 per QALY, and Vallejo‐Torres et al. (2018) found a value of roughly €25,000 for Spain. 1In this context, it is worth noting that a study based on reimbursement decisions in England by National Institute for Health and Clinical Excellence concludes that the incremental cost‐effectiveness ratio (ICER) at which a new technology had a 50% chance of adoption was about £40,000 and an increase of the ICER by £1,000 decreased the odds of being adopted by about 7% (Dakin et al., 2015). Although *k*‐thresholds are typically associated with taking a health care perspective and *v*‐thresholds with a societal perspective, both *k*‐ and *v*‐thresholds can be relevant for both perspectives. From a health care perspective, (an average of) *v* gives an indication as to whether the exogenous health care budget is set optimally (with *v > k* suggesting underspending on health care). Likewise, from a societal perspective, it is important to know, in the case that *v* and *k* are unequal, what the health opportunity costs of spending within the health care sector are, because the adoption of new health care technologies may displace existing technologies. Even if there is no direct displacement within the health care sector, the *k*‐threshold indicates opportunity costs as expanding current interventions rather than implementing new interventions might also be an option.

The objective of this study is to estimate a *k‐*threshold based on the marginal returns to medical care in the Netherlands. Such estimates are necessary to operationalize the full societal decision model used in the Netherlands to decide on the reimbursement of health technologies (Versteegh, Knies, & Brouwer, 2016). Since 2005, cost‐effectiveness has become part and parcel of the Dutch medicine reimbursement system. A range of cost‐effectiveness thresholds between €20,000 and €80,000 have been suggested depending on severity of disease (Zwaap, Knies, van der Meijden, Staal, & van der Heiden, 2015). This range is taken into consideration by the Advisory Committee on Packaging, which advises the National Health Care Institute (ZiN) from a societal perspective on the eligibility of an intervention for inclusion in the benefits package. The threshold range is intended to represent the societal value of a QALY gained (i.e., it is a *v‐*threshold; Brouwer, 2009). Up until now, there have been no studies conducted that aimed to estimate a *k‐*threshold based on the marginal health benefits of Dutch health care. However, Meerding, Polder, de Hollander, and Mackenbach (2007) estimated the average return to health spending between 1953 and 2003 by combining historical data on incidence and mortality for infectious diseases, cancer, and cardiovascular disease (CVD) with information about the year in which specific medical innovations were introduced to construct counterfactuals. On the basis of their analyses, they concluded that the average cost‐effectiveness of Dutch health spending was below €20,000 per QALY gained. Woods, Revill, Sculpher, and Claxton (2016) reported similar estimates (€21,000–28,000) per QALY by extrapolating estimates of the English *k‐*threshold using income elasticity of demand for health, and gross domestic product. To estimate the *k‐*threshold, we will use a Bayesian regression modelling framework, which is in line with the cost‐effectiveness decision framework and allows to incorporate results from previous studies on the impact of health spending on mortality (Claxton, Martin, et al., 2015; Gallet & Doucouliagos, 2017).

Estimating the average of the marginal returns to spending on a bundle of existing interventions is important, yet it is not an easy task: Usually data from randomized controlled trials are not available, as withholding routine interventions from patients who benefit from them would be unethical. Therefore, we make do with observational data and exploit existing variation in health care spending and mortality using econometric methods that address problems of reverse causality and omitted variable bias. When estimating the marginal returns to medical care, we focus on hospital spending on CVD for several reasons. First, much of the impact of increased spending on in‐hospital treatment of CVD will be in the short run, which makes the effects traceable while avoiding strong assumptions about the link between spending and health outcomes. Second, for many interventions in CVD, it is known that by preventing cardiovascular events, they decrease mortality risk (e.g., Cutler, 2007; Ettehad et al., 2016; Nauta et al., 2011; Zijlstra et al., 1999), which emphasizes the strength and relevance of our dataset for the current research question. Third, treatment of CVD is the second‐largest category in terms of hospital care spending, and previous research suggests that in the Netherlands many of the recent gains in life expectancy may have been due to improvements in CVD treatment (Koopman et al., 2011; Mackenbach et al., 2011; Nauta et al., 2011; Vaartjes, O'Flaherty, Capewell, Kappelle, & Bots, 2013). Therefore, estimates using the marginal returns to medical care for this type of care may be estimated more precisely than for other groups of interventions. Finally, the focus on hospital spending is especially relevant for decision making in the Netherlands, as expensive new pharmaceuticals (such as new oncology treatments), which are some of the most debated cases, are primarily financed through reimbursement of hospital expenditures. Currently, annual hospital spending growth in the Netherlands is capped as a result of agreements between the Ministry of Health, hospitals, and medical specialists (1.6% in 2018; Ministerie van Volksgezondheid, 2017). In the absence of specific guidance regarding displacement of technologies, it is likely that if new technologies are reimbursed under hospital budgets, some opportunity costs fall upon CVD patients.

## 2 METHODS

To estimate a *k*‐threshold for hospital care on CVD, we proceed in two steps. First, we estimate the impact of hospital spending on mortality using a Bayesian regression modelling framework. The most important reason for this modelling choice is that the threshold is meant for decision making under uncertainty and that Bayesian estimates facilitate a straightforward interpretation consistent with that aim (Gelman, Carlin, Stern, & Rubin, 2003). Furthermore, the Bayesian framework allows us to investigate the influence of different prior distributions for our key parameter on outcomes. In the second step, we use life tables in combination with quality of life data to convert these estimates into a *k*‐threshold expressed in euros per QALY gained.

### 2.1 Data

**Figure 1:**
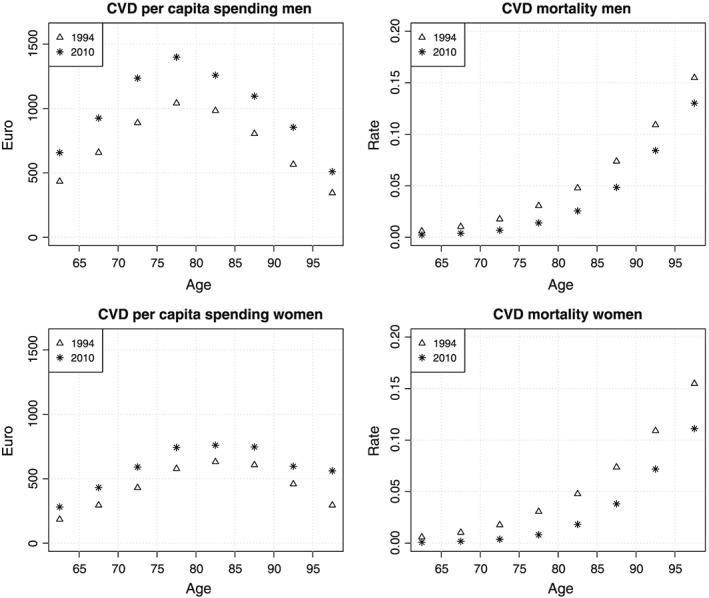
CVD hospital spending and mortality by age and gender in 1994 and 2010. CVD, cardiovascular disease

The data used for the regression model are annual per capita hospital expenditures and mortality rates for CVD for gender‐specific age groups from the years 1994 to 2010 coming from costs of illness studies (Wubulihasimu, Gheorghe, Slobbe, Polder, & Van Baal, 2015). Costs of illness studies have been conducted on a regular basis in the Netherlands using the System of Health Accounts methodology developed by the Organisation for Economic Co‐operation and Development (Heijink, Noethen, Renaud, Koopmanschap, & Polder, 2008; Orosz & Morgan, 2004). In these studies, total hospital costs are uniquely attributed to disease categories using data from the Dutch Hospital Discharge Register, in which almost all hospitals participate, providing almost complete coverage of hospital inpatient admissions in the Netherlands. Costs are allocated to age–gender groups based on day admissions and inpatient admissions, as well as average length of stay of the latter. Cause‐specific mortality rates for CVD were taken from the official mortality register at Statistics Netherlands. In 1994, CVD was responsible for 40% of all deaths in the Netherlands, but by 2010, this percentage had decreased to 30%. Life expectancy at age 65 rose by more than 2 years between 1994 and 2010, more than 1.5 years of which can be attributed to the decrease in CVD mortality. 2Based on our own calculations in which we estimated life expectancy at age 65 by combining CVD mortality rates from 2010 and mortality rates from all other causes from 1994. Hospital spending on CVD grew faster than spending in other disease areas and almost doubled between 1994 and 2010. This can in part be explained by the developments in CVD treatments and technologies, stenting in particular, in the studied period (Simsek, Daemen, & Zijlstra, 2014). From 1994 to 2008, the Netherlands moved from using stents with balloon dilation through to the next‐generation everolimus‐eluting stent. By 2010, hospital spending on CVD accounted for 17% of total hospital spending. Figure 1 displays estimates of per capita hospital spending and mortality by age and gender for 1994 and 2010. It can be seen that CVD spending is higher for men than for women and peaks at about age 80. For both men and women, spending has increased at all ages and mortality has decreased at all ages.

**Figure 2:**
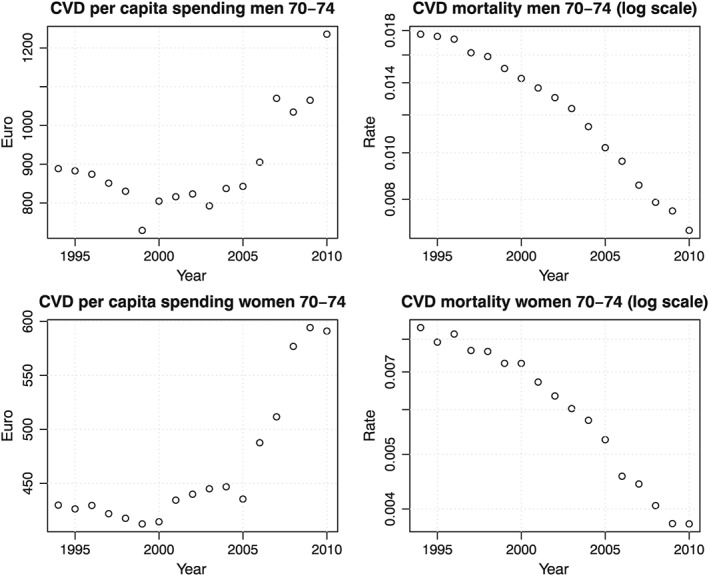
Trends in CVD spending and mortality for the age group 70–74 over time. CVD, cardiovascular disease

To further zoom in on the trends over time, Figure 2 displays trends of CVD spending and mortality for those aged 70–74; for this age group, CVD spending only increased from 2001 onwards and mainly from 2006. CVD mortality decreased in all years but seemed to decrease somewhat faster from 2001 onwards. The spending increase is probably related to a major reform in hospital financing in 2001; it moved from a global budget type of financing to a fee‐for‐service‐like type of financing, which was refined in 2005 to a diagnosis‐based financing scheme (van de Ven & Schut, 2008; Wubulihasimu et al., 2015; Wubulihasimu, Brouwer, & Baal, 2016).

### 2.2 Regression model specification

The outcome of interest in our model is the first difference of log CVD mortality, denoted by *∆* log (*m*_*i*_), where *m* denotes mortality and *i* denotes an index for the number of observations (Year × Age categories × Gender); key covariates are first differences of log CVD spending (denoted by $∆\log\left(c_{i}^{t}\right)$) and 1‐year lagged log CVD spending (denoted by $∆\log\left(c_{i}^{t-1}\right)$). To deal with the omitted variable bias from time‐invariant differences between gender‐specific age groups, for example, age‐ and gender‐specific differences in health, we exploited the panel structure of the data by taking first differences. We used logs for the outcomes and spending measures to deal with skewness and because this means that coefficients may be interpreted as elasticities. Both the current and lagged effects of changes in health spending on mortality were included. A lagged effect of spending on health was included for two reasons. First, research has suggested that improved CVD care might influence mortality rates for more than 1 year (Cutler, 2007; Lagerqvist et al., 2006; Nauta et al., 2011). For those with a severe CVD event, increased quality of care might improve mortality for a longer period after the admission (although the biggest impact is usually right after admission). Furthermore, if the threshold for treatment is lowered, this might mean that less severe patient are admitted too, which might result in preventing possibly lethal CVD events in the subsequent year. Second, because we used calendar year data, a lagged effect captures partly the effects of spending on mortality within 1 year (e.g., persons treated in October still have a lowered CVD mortality risk in May of next year). Since the data are in 5‐year age groups, however, including more than a 1‐year lag of health spending would make less sense as it means linking a large part of spending in one cohort to mortality in another cohort. In addition, the data we use contains mortality and spending of the oldest old (85–89, 90–94, 95+) who are frail and often have co‐morbidities. For these age categories, it is less likely that hospital spending has an impact on mortality more than 1 year after treatment. To adjust for *time‐variant* confounders that affect mortality and spending similarly for all age and gender groups (e.g., the introduction of public smoking bans; de Korte‐de Boer, Kotz, Viechtbauer, et al., 2012), we included year*‐*specific (denoted by *t*) varying intercepts coming from a normal distribution (denoted by *γ*_*t*[*i*]_). To control for trends in CVD mortality not caused by hospital care, such as those caused by trends in lifestyle (e.g., obesity, smoking, and blood pressure; Koopman et al., 2016) that may differ for each age and gender category (denoted by *a*), we also modelled these using varying intercepts coming from a normal distribution (denoted by *τ*_*a*[*i*]_). We centred the distributions of *γ*_*t*_ and *τ*_*a*_ around zero because the model already contained an intercept (denoted by *μ*). This resulted in the following model specification:

(1) $$∆\log\left(m_{i}\right)\sim \text{Normal}\left(\mu +\alpha \;∆\log\left(c_{i}^{t}\right)+\beta \;∆\log\left(c_{i}^{t-1}\right)+\gamma_{t\left[i\right]}+\tau_{a\left[i\right]},\sigma_{m}\right),$$

(2) $$\gamma_{t}∼\text{Normal}\left(0,\sigma_{\gamma }\right),$$

(3) $$\tau_{a}∼\text{Normal}\left(0,\sigma_{\tau }\right).$$

The key identifying assumption is that the variation in mortality trends that cannot be explained by the year‐specific and age/gender‐specific time trends is caused by changes in medical spending, and thus, that the two random intercept distributions and the first differences capture all of the confounders. Equation (1) is the Bayesian equivalent of a basic econometric model specification with first differences, time fixed effects, and age/gender‐specific time trends. Note that the sum of the *α* and *β* parameters, which capture the impact of current and lagged spending on mortality, equals the elasticity of CVD mortality with respect to CVD spending. To complete the Bayesian model specification, prior distributions needed to be defined for all parameters in the model. In our base case analysis, we assumed noninformative/vague priors for all parameters and followed the general advice by Gelman (2006): Prior distributions for all variance parameters (*σ*_*m*_, *σ*_*γ*_, and *σ*_*τ*_) are defined as uniform distributions between 0 and 1. The parameters *μ*, *α*, and *β* are assumed to be normally distributed and in our base case analysis we defined prior normal distributions with mean zero and a variance of 10 for these parameters. The Bayesian regression models were estimated using Markov chain Monte Carlo (MCMC) simulation methods implemented in JAGS (Plummer, 2003). In our MCMC simulations, we used 75,000 iterations of which a burn‐in of 25,000 iterations was discarded, and we used three chains with different starting values in order to ensure that estimates were not influenced by starting values. On the basis of plots for assessing convergence with respect to autocorrelation, we thinned the MCMC output and used only every fourth sample of the MCMC output.

To explore the robustness of our estimates to the most important modelling choices, we estimated several alternative model specifications. In two alternative scenarios, we investigated the impact of more informative priors for *α* based on previous research and varied the number of years included in the sample. In Scenario 1, we used the estimates of a recent meta‐analysis of studies investigating the impact of health spending (total health spending, not CVD specific) on mortality, which estimated an elasticity of −0.13 (−0.20/−0.06) (Gallet & Doucouliagos, 2017). In Scenario 2, we used the outcome elasticity with respect to circulatory problems as estimated by Claxton, Martin, et al. (2015) using 2006/2007 expenditure and 2006/2007/2008 mortality data, which estimated an elasticity of −1.43 (−1.86/−1.00). In Scenario 3, we only used the years 2001–2010 to explore whether the impact of spending on mortality might be stronger when we only include years when hospitals were financed activity based (Wubulihasimu et al., 2016). In Scenario 4, we estimated Equation (1) using a traditional frequentist method by including a set of year and age/gender dummies. Finally, in Scenario 5, we excluded the impact of lagged spending on mortality.

### 2.3 Life table calculations

To translate the elasticity of CVD mortality with respect to CVD spending as estimated by the regression model into a *k*‐threshold, we used life tables linked to quality of life and cost estimates in two different scenarios (see Appendix A for more details on the life table calculations). In the “base case” scenario, we calculated lifetime QALYs and lifetime hospital spending using mortality rates from 2010. In the alternative scenario, we varied CVD mortality resulting from a change in CVD spending: We increased CVD hospital spending by 10% and decreased CVD mortality rates with the product of 0.9 and the elasticity of spending (the sum of *α* and *β*). The *k‐*threshold is then defined as the difference in costs between these scenarios divided by the difference in QALYs. Costs and effects are discounted by 4% and 1.5%, respectively, which is in accordance with Dutch guidelines for economic evaluation (Versteegh et al., 2016).

cWhen translating the life years into QALYs, we assumed that quality of life depends on age and on time to death. The latter was assumed because health losses are usually concentrated in the last phase of life and postponing death via better CVD treatment also postpones health losses often seen at a later age (Gheorghe, Brouwer, & van Baal, 2015; Gheorghe, Picavet, Verschuren, Brouwer, & Baal, 2016). Quality of life estimates by age and time to death attached to the life table were taken from Gheorghe et al. (2015). To include future unrelated medical costs (costs for other diseases besides CVD in life years gained) in our *k‐*threshold estimate, we linked estimates of hospital costs not related to CVD stratified by disease, age, sex, and time to death, from PAID 1.1 (www.imta.nl/paid; van Baal et al., 2011), to the life tables.

Incremental costs and QALYs due to increased CVD spending were calculated for the same age categories as used in the data (60–64, 65–69, …, 95+) for both genders and then aggregated and weighted by population size. To account for uncertainty in costs and effects, we repeated this procedure for all values of the joint posterior distribution of *α* and *β* that were estimated. To investigate the impact of our assumptions with respect to quality of life, we also estimated costs per life year gained. Note that if we were to employ this methodology and attribute the entire decrease in CVD mortality in the period 1994–2010 to hospital care, this would result in a threshold estimate of €4,500 per QALY gained. Here, we applied the life table methodology as explained in Appendix A, using CVD spending and CVD mortality from 1994 in the baseline scenario, and CVD spending and CVD mortality from 2010 in the alternative scenario.

## 3 RESULTS

**Figure 3:**
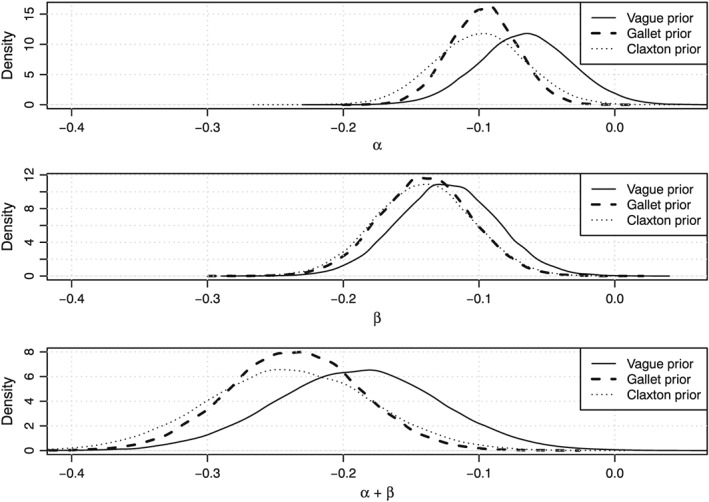
Posterior distributions of *α* (top panel), *β* (middle panel), and the sum of *α* and *β* (bottom panel) for various prior distributions of *α*

Figure 3 displays posterior distributions of *α*, *β*, and the sum of *α* and *β* for various prior distributions of *α* (the base case model and Scenarios 1 and 2). It can be observed that the instantaneous impact of spending on mortality (*α*) in our study is much smaller than the estimates of Claxton, Martin, et al. (2015) and Gallet and Doucouliagos (2017). Furthermore, the lagged impact (*β*) of spending on mortality is bigger than the instantaneous impact. Taken together (*α* + *β*), the impact of spending on mortality is bigger than estimated by Gallet and Doucouliagos in their meta‐analysis but still much smaller than the impact of spending on mortality estimated by Claxton, Martin, et al. (Gallet & Doucouliagos, 2017). Furthermore, Figure 3 suggests that a more informative prior, that is, one based on previous studies, shifts the posterior of *α* to the left. Figure 3 also suggests that the prior based on the study by Gallet and Doucouliagos creates a much smaller variance of the posterior. Additionally, Figure 3 shows that the estimates of *α* and *β* are positively correlated, as the estimates of *β* are also lower when using the informative priors for *α*.

Table 1 provides a summary of the regression results, the combined effect *α* + *β*, and the corresponding estimates of the threshold in terms of costs per QALY gained and costs per life year gained. In the base case scenario, the threshold is estimated to equal €41,000 and the costs per life year gained equal €30,000. The difference between costs per life year gained and costs per QALY gained can be explained by the fact that, regardless of the postponement of quality of life losses, life years gained are not lived in perfect health. With the use of the base case estimates, a back‐of‐the‐envelope calculation reveals that 10% to 15% of the increase in life expectancy at age 65 due to CVD can be attributed to hospital care spending growth.

**Table 1:** Mean posterior parameter estimates with 95% credible intervals of the regression between brackets

Scenario	*α*	*β*	*α* + *β*	Cost per QALY gained	Cost per life year gained
Base case scenario	−0.065 (−0.133/0.001)	−0.124 (−0.194/−0.052)	−0.190 (−0.309/−0.069)	41,000 (25,900/110,400)	30,000 (18,800/80,400)
Scenario 1 (prior *α* based on Gallet and Doucouliagos)	−0.096 (−0.150/−0.048)	−0.139 (−0.205/−0.072)	−0.236 (−0.331/−0.140)	33,400 (24,200/55,200)	24,300 (17,600/40,200)
Scenario 2 (prior *α* based on Claxton et al.)	−0.100 (−0.166/−0.034)	−0.141 (−0.213/−0.071)	−0.241 (−0.360/−0.123)	32,700 (22,400/62,400)	23,800 (16,300/45,400)
Scenario 3 (2001–2010 data only)	−0.088 (−0.181/0.005)	−0.117 (−0.202/−0.031)	−0.204 (−0.355/−0.054)	38,400 (22,700/141,500)	28,000 (16,500/103,000)
Scenario 4 (frequentist approach)	−0.066 (−0.134/0.002)	−0.125 (−0.198/−0.053)	−0.191 (−0.312/−0.070)	40,800 (25,600/109,100)	29,700 (18,600/79,500)
Scenario 5 (excluding lagged effect)	−0.013 (−0.070/0.047)	NA	NA	690,000 (108.800/−154,500)	502,500 (79,500/−112,600)

*Note*. Costs per QALY and life year calculated by dividing mean costs by mean effects. 95% credible intervals calculated by setting the sum of *α* + *β* at 0.025 and 0.975 quantile. QALY, quality‐adjusted life year.

From Table 1, it is clear that in the Alternative Scenarios 1 and 2, the ICER estimates are lower because the estimates of *α* and *β* become more negative with a more informative prior. If we use the estimates of the traditional frequentist estimation procedure (Scenario 4), in which we modelled the age‐, gender‐, and year‐specific effects using fixed effects rather than random effects, the results are almost identical to those of the base case scenario—also in terms of credible intervals. It appears that the use of vague priors (which increase the credible intervals) is counterbalanced by the use of random effects. In Scenario 3, where we used a subset of years included in the panel, the estimates of the impact of spending on mortality are only slightly higher. This seems to indicate that the relation between spending and mortality has not changed that much as a consequence of the hospital payment reform. However, this does not imply that the payment reforms did not have any impact, as they probably triggered the increase in health spending. Excluding the impact of lagged spending (Scenario 5) lowers the impact of current spending on mortality because spending change and lagged change in health spending are negatively correlated. Given the absence of lagged spending on mortality and a reduced instantaneous effect of spending on mortality, the threshold rises dramatically in Scenario 5. We prefer the models that include lagged spending because the coefficient *β* has a strong impact in all models, which corroborates that CVD‐related spending has a positive impact on mortality in the subsequent year too.

**Figure 4:**
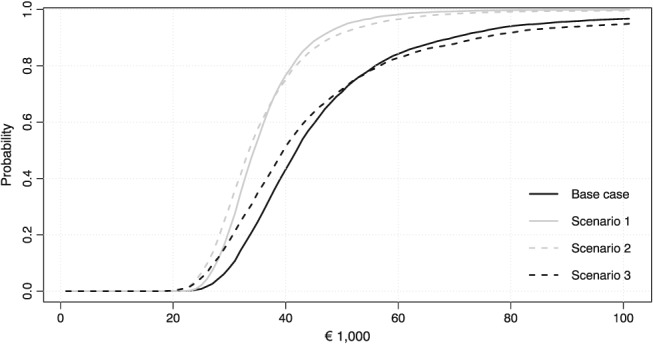
Curves indicating the probability that the *k*‐threshold is below a certain monetary value for the base case scenario and Scenarios 1, 2, and 3 (NB: Scenario 4 is not displayed because it almost overlaps with the base case scenario. Scenario 5 is not displayed because the probability that the *k*‐threshold is below a certain monetary value remains low for all monetary values)

To facilitate a better understanding of the uncertainty surrounding the *k‐*threshold estimates, Figure 4 displays probabilities that the estimated threshold lies below a certain monetary value (van Hout, Al, Gordon, & Rutten, 1994). Here, again, the relevance of the prior distributions becomes clear as in Scenarios 1 and 2 the curve shifts to the left and probabilities converge to 1 for higher monetary values.

## 4 CONCLUSION AND DISCUSSION

In this paper, we estimated the marginal returns to (CVD) medical care spending in the Netherlands using a Bayesian approach and translated these into a *k‐*threshold. The Bayesian approach enabled us to investigate the impact of various prior distributions for key parameters in our model. These priors proved to have a large impact on the resulting threshold estimate and to a lesser extent also on its uncertainty. In our base case estimate, we employed a noninformative prior, which resulted in an estimate of 41,000 per QALY gained. In our sensitivity analyses, we illustrated how the incorporation of prior evidence in the estimation pushed the estimates downwards. Furthermore, our sensitivity analysis indicated that excluding a lagged effect of spending on mortality increases the *k‐*threshold considerably.

Our estimate of the marginal returns to CVD spending is based on variation at the margins of different age–gender groups but is ultimately an average treatment effect across all these different age–gender groups. Furthermore, this estimate represents the average impact of multiple interventions, which are displaced or expanded at these margins. Interventions that might have caused the estimated reductions in CVD mortality include primary percutaneous coronary intervention, improved antithrombotic and anticoagulant therapy, and tailored treatment with selective coronary revascularization, which were increasingly common during the study period and are known to decrease mortality for over a longer period of time (Nauta et al., 2011). Our estimates of the marginal returns to medical spending are higher than the estimates in the study of Meerding et al. (2007). The most important reason for this is probably that our study focused on estimating the marginal rather than average returns to medical spending over a much longer period. Our study also used *more recent data*, and it seems plausible that returns to medical spending have fallen over time if new medical technologies are priced at the margin. Second, we focused on *hospital care spending only* whereas Meerding et al. used broader measures of medical spending including ambulatory care and medication. As general practitioners prescribe much of the CVD medication that has been shown to be lifesaving such as statins and beta‐blockers, it is not improbable that in these sectors returns to medical spending are higher than in hospital care. If this were true, it might also help explain why our *k‐*threshold is higher than those obtained by Claxton, Martin, et al. (2015) 3Note that Claxton also estimated a threshold specific for circulatory problems using 2006/2007 expenditure and 2006/2007/2008 mortality data, which equalled £5,631, which is even lower than their overall threshold. It is this threshold that is more directly comparable with our estimate. and Vallejo‐Torres et al. (2018) who included spending on ambulatory care. The fact that the lagged impact of spending on mortality is bigger than the instantaneous impact in our analysis was unexpected. It suggests that the effect of spending on mortality is to a large extent achieved by lowering the treatment threshold. Increasing volume by treating milder patients in hospital for CVD might prevent cardiovascular events but does not have instantaneous effect as would be the case if the quality of treatment improves for severe patients (e.g., patients admitted for a stroke and acute myocardial infarction). This broadening of the indication area is also seen in practice as there has been a much larger growth in day‐care admissions than in clinical admissions (Central Bureau for Statistics, 2017). Another possible explanation might be that we are capturing some effects of improved out‐of‐hospital care. Our findings are in line with those of a previous study that used various data sources to attribute changes in CVD mortality, which argued that 36% was attributable to medical care as our estimates suggest that 10% to 15% is due to hospital care (Koopman et al., 2011).

Much like any study, our study has some limitations, which may have resulted in a biased estimate of the *k‐*threshold. First, although we limit the influence of omitted variables by exploiting the panel structure of the data, by including first differences and time‐specific and age/gender‐specific random intercepts, we cannot establish whether we have successfully controlled for all potentially relevant confounders. An omitted variable that may potentially have a large impact on the results is the use of prescription drugs that are prescribed for CVD but that are not paid for through the hospital budget. As some of these drugs work well, and as the use of these drugs increased during the study period, it would mean that we overestimated the impact of hospital spending on CVD‐related mortality. However, descriptive statistics reveal that the trend in CVD prescription drugs use was much less strong than the trends in CVD‐related hospital spending and mortality, suggesting that the impact of trends in prescription drug use may have been limited (GIPdatabank, 2017). In contrast to previous work (Claxton, Martin, et al., 2015; Vallejo‐Torres et al., 2018), we only controlled for health status indirectly by first differencing the data and using random effects. To what extent this results in an overestimate or underestimate of the threshold is difficult to assess. Second, we have not been able to deal with—or test for—potential reverse causality, which may stem from the fact that most spending occurs in the last years before death, implying that (conditional on age) lower mortality rates should be accompanied by lower health care spending (van Baal & Wong, 2012). Hence, reverse causality would mean that our estimate of the impact of health spending on mortality is an underestimate and the *k*‐threshold estimate is an overestimate. However, the impact of reverse causality is probably limited as the time to death effect is not as strong for CVD as it is for other diseases such as cancer (Wong, van Baal, Boshuizen, & Polder, 2011). Furthermore, in this context, it should be noted that the impact of any linear autonomous trend in age‐ and gender‐specific mortality on spending is picked up by the random effects included in our model. A method accounting for unobserved confounders and reverse causality would be an instrumental variable analysis, which has been used in previous work (Claxton, Martin, et al., 2015; Vallejo‐Torres et al., 2018). However, finding relevant and valid instruments is not an easy task. Given that we are dealing with panel data using lagged independent variables as instruments as proposed by Arellano–Bond method might be worth considering (Arellano & Bond, 1991). However, the Arellano–Bond method also carries its own assumptions that need to be satisfied. Third, we did not directly estimate an effect of health care spending on quality of life. Instead, we assume a stable relationship between age, gender, mortality risk, and quality of life, which is estimated in a previous study (Gheorghe et al., 2015, 2016). This assumption recognizes that quality of life losses are postponed to a later age if death is postponed. Using this relationship between mortality and quality of life results in improved quality of life profiles by age if mortality decreases. The study by Claxton, Martin, et al. (2015) instead assumed the same proportional impact of spending on quality of life as on mortality, arriving at the conclusion that 30% of the QALY gains due to CVD treatment are due to quality of life improvements. If we had adopted this assumption, the impact of spending on quality of life would have been larger and the threshold would have been lower as only 5% of the QALY gains in our analysis are due to quality of life improvements. Note that in contrast to previous studies, we explicitly included future unrelated medical costs in the estimation of the *k*‐threshold (van Baal, Meltzer, & Brouwer, 2016). It should be noted however that the impact was really small given that a large part of hospital spending is centred in the last year of life (and costs in the last year of life decrease at higher ages). Finally, in our translation of our regression estimates to a *k*‐threshold, we used population mortality rates to calculate life years gained, which may have resulted in an overestimate considering that CVD patients are less healthy than the average population. All in all, some limitations of our analyses suggest that we might have overestimated the threshold, whereas other limitations indicate an underestimate of the threshold. Although it is not possible to weigh the sizes of either, the presence of bias in both directions (i.e., overerestimation and underestimation) at least allows for the possibility that they cancel each other out, or that the current estimate is a fair point estimate for the mean of the distribution of potential thresholds. Future research is needed to quantify the magnitude of these different potential biases.

Our results are relevant for Dutch policy regarding the reimbursement of new expensive drugs. In the Netherlands, where many of these new drugs are provided and paid for within a capped hospital budget, they are directly competing for funds with other hospital‐based activities funded from that same budget. Currently, the Dutch ministry negotiates over prices of new medication using the €20,000 to €80,000 *v‐*threshold range, with higher thresholds for more severe diseases (expressed in terms of proportional shortfall; Stolk, van Donselaar, Brouwer, & van Busschbach, 2004). Adopting an €80,000 per QALY threshold could result in health losses if opportunity costs fall within CVD care and these are not accounted for. Whether or not such a health loss is justified also depends on the relative value attached to health gains in different circumstances (Bobinac, van Exel, Rutten, & Brouwer, 2012). This issue of relative value of health gains in relation to empirical estimates of marginal cost‐effectiveness of current care is an understudied topic.

Although the *k‐*threshold estimates presented in this study are imprecise and surrounded by a considerable amount of uncertainty, it may well be that the opportunity costs within the health care sector are lower than current *v‐*thresholds (Zwaap et al., 2015). In all scenarios, there is a high probability (>0.9) that the opportunity costs within the health care sector are below €80,000 per QALY gained. We do emphasize that in the Dutch context the €80,000 per QALY *v‐*threshold is reserved only for interventions treating patients with the highest burden of disease. For CVD, the relevant average threshold in the current framework appears to be €50,000 per QALY (Corro Ramos et al., 2017), which is still higher than the estimated *k*. More generally, even if opportunity costs do not fall within CVD care or even fall outside medical care, the €41,000 estimate still represents an estimate of opportunity costs for all new investment opportunities in health care, assuming that CVD care can be expanded to a wider patient group with a similar cost‐effectiveness, for example, through slightly less stringent indication setting. In price negotiations, these estimates (and estimates for other disease areas from future research) may be used as relevant reference points, as new technologies compete for funding with old technologies that are represented in these estimates. Given that the highest thresholds now used in the Netherlands (of €50,000 and €80,000 per QALY depending on disease severity) are higher than our estimate of (some of) the opportunity costs of spending, this is relevant information to include in negotiations and decision making. New in‐hospital technologies will have to be paid for from fixed hospital budgets, so these estimates of health opportunity costs, which can and need to be improved and expanded, are relevant as a first estimate for both funding and pricing decisions.

Given the relevance of *k‐*thresholds for Dutch policy, and in making the full decision‐making framework completely operational (Claxton et al., 2011), future research should also focus on estimating the returns of medical spending in other disease areas and other health care sectors. However, this might be even more challenging than for hospital CVD care as for many diseases/health care sectors the impact is more likely to be on quality of life rather than mortality. Moreover, there can be a longer time lag between spending and outcomes. For diseases where it is more likely that there is an instantaneous impact of spending on mortality such as cancer, problems of reverse causality are severe given that spending on cancer is heavily concentrated in the last year of life (Wong et al., 2011). More generally, research on the impact of spending on quality of life is needed, which could be informed by the many quality measurement‐related initiatives in the Netherlands and abroad. Furthermore, future research should try to answer to what extent new technologies actually displace older ones, and if so, through what mechanisms. For this, quantitative research alone is not sufficient, and qualitative research may shed light on how the process of technology adoption and displacement occurs in practice (Schaffer, Sussex, Hughes, & Devlin, 2016).

In conclusion, this is the first attempt at laying an empirical foundation for a *k‐*threshold value for the Netherlands. On the basis of the marginal returns for CVD hospital care, we estimated the *k‐*threshold to equal €41,000 per QALY gained. Although this estimate is surrounded by considerable uncertainty and our analyses highlight the need for better data, this *k*‐threshold estimate may serve as a reference value to be used in the full decision‐making framework in the Netherlands. The methods and data we employed might inspire research on *k‐*thresholds in the Netherlands and other countries.

## APPENDIX A

### LIFE TABLE CALCULATIONS

The following formulas were used in the life tables:

$$Q_{b,g}=\sum_{a=b}^{100}\left(1.015^{a-b}\times q_{a,g}\times \prod_{b}^{a}e^{-m_{a,g}^{d}-m_{a,g}^{o}}\right)\;C_{b,g}=\sum_{a=b}^{100}\left(1.04^{a-b}\left[c_{a,g}^{d}+c_{a,g}^{o}\right]\times \prod_{b}^{a}e^{-m_{a,g}^{d}-m_{a,g}^{o}}\right),$$

Where:

*Q*_*b*,*g*_: lifetime expected discounted QALYs for a person at age *b* and gender *g*
*C*_*b*,*g*_: lifetime expected discounted hospital costs for a person at age *b* and gender *g*
*q*_*a*,*g*_: average quality of life at age *a* gender *g*
$m_{a,g}^{d}$: mortality rate for cardiovascular disease (CVD) at age *a* gender *g*
$m_{a,g}^{o}$: other cause mortality rate for CVD at age *a* gender *g*
$c_{a,g}^{d}$: per capita hospital spending for CVD at age *a* gender *g*
$c_{a,g}^{o}$: per capita hospital spending for other diseases at age *a* gender *g*.

Indices for years are suppressed as all data were taken for the year 2010. To translate the life years into QALYs, we assumed that quality of life depends on age and time to death. The latter is done because health losses are usually centred in the last phase of life. Quality of life (denoted by *q*) values were taken from Gheorghe et al. (2015) who estimated quality of life by age and time to death by linking health survey data containing the SF‐12 (from which the SF‐6D was derived) to the death registry. Consequently, if increased spending on CVD decreases mortality at a given age, it also improves quality of life at that age:

$$q_{a,g}=\left(m_{a,g}^{d}+m_{a,g}^{o}\right)\times q_{a,g|m=1}+\left(1-m_{a,g}^{d}-m_{a,g}^{o}\right)\times q_{a,g|m=0},$$

where *q*_*a*,*g*|*m*=1_ indicates quality of life at age *a* gender *g* conditional on the fact that this person dies at age *a* (the last year of life), and *q*_*a*,*g*|*m*=0_ indicates quality of life at age *a* gender *g* conditional on not dying at age *a*. For hospital costs not related to CVD, we also make a distinction between the last of life (decedent costs) and other years of life (survivor costs):

$$c_{a,g}^{o}=\left(m_{a,g}^{d}+m_{a,g}^{o}\right)\times c_{a,g|m=1}^{o}+\left(1-m_{a,g}^{d}-m_{a,g}^{o}\right)\times c_{a,g|m=0}^{o}.$$

In the base case scenario, we used CVD mortality rates, other cause mortality rates, and CVD hospital spending values from the year 2010. In the alternative scenarios, we used increased CVD hospital by 10% and decreased CVD mortality rates with the product of 0.9 and the elasticity of spending (the sum of *α* and *β*). Quality of life and hospital (unrelated) spending then changed as a result of changing mortality rates. The *k‐*threshold is then defined as the difference in costs between these scenarios divided by the difference in QALYs aggregated and weighted by population size by age and gender (denoted by *pop*_*b*,*g*_). The threshold estimate was calculated in the following manner:

$$\text{ICER}=\frac{\sum_{g}\sum_{b=65}^{100}{\mathrm{pop}}_{b,g}\times \left(C\prime_{b,g}-C_{b,g}\right)}{\sum_{g}\sum_{b=65}^{100}{\mathrm{pop}}_{a,g}\times \left(Q\prime_{b,g}-Q_{b,g}\right)},$$

where *C*_*b*,*g*_ and *Q*_*b*,*g*_ indicate lifetime QALYs and costs in the base case scenario and *C*′_*b*, *g*_ and *Q*′_*b*, *g*_ indicate lifetime QALYs and costs in the alternative scenario in which mortality and spending are changed.
